# Suicidal behavior among farmers in the semiarid Northeast region: a case-control study

**DOI:** 10.47626/1679-4435-2025-1414

**Published:** 2025-11-17

**Authors:** Emelynne Gabrielly de Oliveira Santos, Isabelle Ribeiro Barbosa

**Affiliations:** 1 Graduate Program in Public Health, Universidade Federal do Rio Grande do Norte, Natal, RN, Brazil.; 2 Trairi School of Health Sciences, Universidade Federal do Rio Grande do Norte, Santa Cruz, RN, Brazil.

**Keywords:** self-injurious behavior, rural health, pesticide exposure, mental disorders, comportamento autodestrutivo, saúde da população rural, exposição à praguicidas, transtornos mentais

## Abstract

**Introduction:**

Suicidal behavior is a complex, multifactorial phenomenon resulting from the interaction of philosophical, psychological, biological, and social factors. Agriculture is an occupation in which individuals are particularly vulnerable to mental distress and suicidal behavior.

**Objectives:**

To analyze factors associated with suicidal behavior among farmers living in the semiarid region of northeastern Brazil.

**Methods:**

This case-control study was conducted between 2019 and 2020 with 450 farmers from the city of Caicó, Rio Grande do Norte. The final sample included 62 cases and 288 controls. Suicidal behavior was assessed using the Beck Scale for Suicide Ideation. Associations were analyzed with sociodemographic, health, income, work, and alcohol abuse variables.

**Results:**

A significant positive association was observed between suicidal behavior and having a family history of mental disorder (odds ratio = 2.76), having a common mental disorder (odds ratio = 4.25), having previously received mental health treatment (odds ratio = 2.36), performing wage or temporary work (odds ratio = 3.49), and having experienced pesticide poisoning (odds ratio = 4.94).

**Conclusions:**

Work conditions, mental health factors, and pesticide poisoning are associated with suicidal behavior among farmers in the semiarid region.

## Abstract (pt)

**Introdução:**

O comportamento suicida configura-se como um fenômeno complexo e multifatorial, que resulta da interação de fatores filosóficos, psicológicos, biológicos e sociais. A agricultura é uma atividade na qual os indivíduos estão vulneráveis ao sofrimento mental e ao comportamento suicida.

**Objetivos:**

Analisar fatores associados ao comportamento suicida em agricultores do semiárido nordestino.

**Métodos:**

Estudo caso-controle, cuja coleta de dados se deu entre 2019 e 2020, com 450 agricultores do município de Caicó, no Rio Grande do Norte. O tamanho da amostra final foi definido em 62 casos e 288 controles. Avaliou-se o comportamento suicida através do Inventário de Ideação Suicida de Beck, e foram analisadas as associações com variáveis sociodemográficas, de saúde, renda, trabalho e uso abusivo do álcool.

**Resultados:**

Foi observada associação positiva e significativa entre o comportamento suicida e ter diagnóstico de transtorno mental na família (*odds ratio* = 2,76), ter transtorno mental comum (*odds ratio* = 4,25), ter realizado previamente tratamento para saúde mental (*odds ratio* = 2,36), realizar trabalho assalariado ou temporário (*odds ratio* = 3,49) e ter tido intoxicação por agrotóxicos (*odds ratio* = 4,94).

**Conclusões:**

Este estudo indicou que os aspectos de trabalho, de saúde mental e intoxicação por agrotóxicos estão associados com o comportamento suicida em agricultores.

## INTRODUCTION

Agriculture exposes individuals to multiple vulnerabilities related to mental health due to the fears and uncertainties that permeate both rural life and agricultural work itself.^1^ Drought, limited access to health services, and frequent pesticide exposure contribute to psychological distress in this population. These conditions may be associated with suicidal behavior, which includes suicidal ideation, planning, attempts, and the act itself.^2^

Suicide is among the leading causes of mortality worldwide, with an estimated global rate of 9.0 per 100,000 inhabitants. Although most countries experienced a substantial reduction in suicides between 2000 and 2019 (a 36% global decrease), the Region of the Americas moved in the opposite direction, with a 17% increase over the same period. Brazil, in particular, has stood out for some of the largest increases since 2000; between 2010 and 2019, the national rate rose by 43%, reaching 6.7 per 100,000 inhabitants.^3^

Studies conducted in different world regions have reported high prevalences of suicidal behavior among farmers.^4^ In countries such as Nigeria and Uganda, for example, the prevalence of suicidal ideation among farmers reached 29.7% and 21.3%, respectively.^5^ Research indicates that the main factors associated with suicidal behavior are primarily related to mental health and pesticide exposure in rural working environments.^6^

In Brazil, a number of studies have identified the prevalence of suicidal behavior and its association with occupational and environmental factors. A study in the state of Piauí, conducted among residents of rural settlements, found a 13.4% prevalence of suicidal ideation.^7^ Another, in Minas Gerais, showed that farmers exposed to pesticides — compared with those engaged in agroecological practices — had higher odds of suicidal ideation, problematic alcohol use, and prior episodes of acute pesticide poisoning.^8^

Ethnoracial, cultural, and behavioral issues are also important indicators for this outcome. Low income, limited social support, isolation, and loneliness contribute to a range of emotional problems, producing or exacerbating behavioral and mental disorders associated with suicidality. Given the complexity of phenomena linked to suicide, a broad, multidimensional approach is required, incorporating aspects from different domains of life—work among them.^9^

In rural settings, ways of life and working conditions can mask adverse realities marked by socioeconomic vulnerabilities, limited access to education, precarious labor conditions, and deficiencies in health services — especially in mental health. These factors may increase the risk of suicidal behavior in this population, particularly among farmers. Moreover, substantial disparities exist between suicide mortality rates in urban versus rural areas, and agriculture is recognized as an important occupational risk factor for this outcome.^10,11^

Studies conducted in the state of Rio Grande do Norte (RN) have identified a relationship among suicide, rural populations, and agricultural work, evidenced by high mortality rates in this group. Botega et al.^12^ highlighted that the city of Caicó, located in the interior of the state, ranked third among the 20 Brazilian cities with at least 50,000 inhabitants that had the highest suicide coefficients in the country from 2006 to 2010. Additionally, Lins^13^ demonstrated that Caicó had the highest suicide rate among farmers between 2000 and 2015.

Suicidal behavior can thus be understood as a broad, complex process in which farmers constitute one of the high-risk occupational groups. Scientific production and the strengthening of strategies to screen farmers for suicidal behavior are essential to enable early detection of psychological distress and suicide risk, thereby contributing to decrease suicide mortality in this population.

Policymakers and health professionals should consider developing and offering mental health education programs for farmers and those who work closely with them to identify symptoms of mental health problems and facilitate attitude change. Expanding access to health care must be a priority in rural areas, and clinicians should be familiar with the stressors farmers face so they can ask about work-life balance and better assess farmers’ mental health and suicide risk.^14^

As suicide attempts and deaths often originate from suicidal ideation, which are a key indicator in suicide prevention, it is important to understand the scale of this issue and the factors associated with it among farmers in Caicó, who are at an increased risk of suicide due to vulnerabilities and exposures widely reported in the literature.

This study is based on the hypothesis that domains of life, health, and work — such as the presence of mental disorders and pesticide use — are associated with aspects of suicidal behavior. Accordingly, the objective was to analyze factors associated with suicidal behavior among farmers in the city of Caicó, state of RN.

## METHODS

### STUDY DESIGN AND POPULATION

This was an observational case-control study conducted in the municipality of Caicó, state of RN, Brazil. The target population comprised farmers registered with the Rural Workers’ Union of Family Farmers (STRAAF, in Portuguese). Data were collected from August 2019 to March 2020.

### SAMPLE SIZE

The sample size was estimated assuming an expected odds ratio (OR) of 2.5 and a 20% exposure prevalence among controls. We adopted a 5% significance level (α = 0.05), 80% statistical power (1 - β = 0.80), and a 4:1 control-to-case ratio. To compensate for refusals and losses, 15% was added to the calculated total. The final sample consisted of 62 cases and 288 controls.

### SELECTION OF CASES AND CONTROLS

Inclusion criteria were STRAAF registration and age ≥ 18 years. The total eligible population was approximately 2,000 farmers. Simple random sampling was used, and household interviews were conducted by trained interviewers after participants signed the informed consent form (ICF).

Cases were defined as individuals who presented suicidal ideation or a prior suicide attempt, as identified by the Beck Scale for Suicide Ideation.^15^ The scale comprises 21 items: the first 19 assess the severity of ideation, attitudes, and suicidal plans, whereas the last two capture information on previous attempts and intent to die. Participants were classified as cases if they gave a nonzero response to items 1, 2, 3, 4, 5, or 20.

Controls were individuals from the same population, aged ≥ 18 years, who scored zero on the items listed above. Controls were matched to cases by sex and age group (18-39, 40-59, and ≥ 60 years) in a 4:1 ratio.

### STUDY VARIABLES AND DATA COLLECTION INSTRUMENTS

Independent variables were grouped into three categories — sociodemographic, health, and work-related domains. Sociodemographic variables included marital status, skin color, education, religion, income, place of residence, household size, and access to sanitation. Health-related variables included self-rated health; family history of mental disorder; presence of a common mental disorder; prior mental health treatment; smoking; alcohol abuse; perceived access to health services; and coverage by primary care. Alcohol abuse was assessed using the CAGE (Cut down, Annoyed by criticism, Guilty, and Eye-opener) questionnaire.^16^

The presence of a common mental disorder was assessed using the Self-Reporting Questionnaire, applying the cutoffs reported by De Jesus & Williams^17^; for participants aged > 60 years, the cutoff followed Scazufca et al.^18^

Work-related variables included current employment status; daily working hours; employment relationship; access to credit; indebtedness; production loss; contact with pesticides; use of personal protective equipment (PPE); prior pesticide poisoning; and the stage of farming activities in which exposure occurred.

### DATA ANALYSIS

We first conducted descriptive analyses of variables by case and control groups and compared proportions using the chi-square (χ2) test.

In the bivariate stage, we estimated odds ratios (ORs) with 95%CIs. Variables with p < 0.20 were entered into a multivariable model using backward stepwise logistic regression, with statistical significance set at p < 0.05. Adjusted associations are reported as adjusted ORs with 95%CIs. Model fit was assessed using the Hosmer-Lemeshow goodness-of-fit test and Nagelkerke’s pseudo R^2^. The final model retained only variables that remained statistically significant, with adjustment for sex, age, skin color, and income. Analyses were performed in Stata 13 (StataCorp, College Station, TX, USA).

### ETHICAL CONSIDERATIONS

This study was approved by the Research Ethics Committee of Onofre Lopes University Hospital, Universidade Federal do Rio Grande do Norte CAAE 15532919.5.0000.5292; July 5, 2019). It complied with Brazilian National Health Council Resolution No. 466/2012. All participants provided written ICFs prior to the interviews.

## RESULTS

The distribution of socioeconomic variables was similar between the case and control groups, with no statistically significant differences (Table 1). Age distribution was uniform (p = 0.862), with 33.68% of participants in the control group and 37.10% in the case group aged 18-39 years, and 19.44% and 19.35%, respectively, aged 60 years or older. Regarding sex, a similar pattern was observed (p = 0.563): 47.57% of individuals in the control group and 51.61% in the case group were female.

**Table 1:** Distribution of control and case groups by socioeconomic and demographic characteristics among farmers in Caicó, Rio Grande do Norte, Brazil

Variables	Controls (n = 288) n (%)	Cases (n = 62) n (%)	p-value	Crude OR	95%CI	p-value
Marital status						
Married	204 (70.83)	47 (75.81)	0.700	1.00		
Single/divorced	64 (22.22)	12 (19.35)		0.81	0.40-1.62	0.560
Widowed	20 (6.94)	3 (4.84)		0.65	0.18-2.28	0.502
Skin color						
White/other	138 (47.92)	25 (40.32)	0.277	1.00		
Black or Brown	150 (52.08)	37 (59.68)		1.36	0.77-2.37	0.278
Education						
Secondary education and higher education	43 (14.93)	7 (11.29)	0.300	1.00		
Primary education	170 (59.03)	33 (53.23)		1.19	0.49-2.87	0.696
No formal schooling	75 (26.04)	22 (35.48)		1.80	0.71-4.56	0.214
Religion						
Yes	206 (71.53)	48 (77.42)	0.346	1.00		
No	82 (28.47)	14 (22.58)		0.73	0.38-1.40	0.347
Income (Brazilian minimum monthly salary)						
< 1	84 (29.17)	18 (29.03)	0.983	0.99	0.54-1.81	0.983
> 1	204 (70.83)	44 (70.97)		1.00		
Place of residence						
Urban	16 (5.56)	5 (8.06)	0.450	1.00		
Rural	272 (94.44)	57 (91.94)		0.67	0.23-1.90	0.453
Household size (residents)						
0-2	55 (19.10)	15 (24.19)	0.466	1.50	0.74-3.01	0.255
3-4	154 (53.47)	28 (45.16)		1.00		
> 5	79 (27.43)	19 (30.65)		1.32	0.69-2.51	0.394
Access to sanitation						
Yes	61 (21.18)	17 (27.42)	0.284	1.00		
No	227 (78.82)	45 (72.58)		0.71	0.38-1.32	0.286

OR = odds ratio.

In the bivariate analysis, suicidal behavior was significantly associated with the following variables: family history of mental disorder (p < 0.005), presence of a common mental disorder (p = 0.003), prior mental health treatment (p < 0.005), smoking (p = 0.008), and alcohol abuse (p = 0.008) (Table 2). Associations were also observed with employment relationship (p = 0.001), access to credit (p = 0.066), and history of pesticide poisoning (p = 0.006) (Table 3).

**Table 2:** Distribution of control and case groups by health characteristics among farmers in Caicó, Rio Grande do Norte, Brazil

Variables	Controls (n = 288) n (%)	Cases (n = 62) n (%)	p-value	Crude OR	95%CI	p-value
Self-rated health						
Very good/good	153 (53.13)	32 (51.61)	0.829	1.00	
Fair/poor/very poor	135 (46.88)	30 (48.39)		1.06	0.61-1.84	0.829
Family diagnosis of mental disorder						
No	141 (48.96)	13 (20.97)	< 0.005	1.00	
Yes	147 (51.04)	49 (79.03)		3.61	1.88-6.95	< 0.005
Common mental disorder						
No	120 (41.67)	13 (20.97)	0.002	1.00	
Yes	168 (58.33)	49 (79.03)		2.69	1.39-5.18	0.003
Prior mental health treatment						
No	218 (75.69)	28 (45.16)	< 0.005	1.00		
Yes	70 (24.31)	34 (54.84)		3.78	2.14-6.67	< 0.005
Smoking						
No	224 (77.78)	38 (61.29)	0.007	1.00		
Yes	64 (22.22)	24 (38.71)		2.21	1.23-3.95	0.008
Alcohol abuse						
No	204 (70.83)	33 (53.23)	0.007	1.00		
Yes	84 (29.17)	29 (46.77)		2.13	1.21-3.73	0.008
Access to health services						
No	80 (27.78)	11 (17.74)	0.102	0.56	0.27-1.13	0.106
Yes	208 (72.22)	51 (82.26)		1.00		
Primary health care coverage						
No	13 (4.51)	6 (9.68)	0.104	2.26	0.82-6.21	0.112
Yes	275 (95.49)	56 (90.32)		1.00		

OR = odds ratio.

**Table 3:** Distribution of control and case groups by occupational characteristics among farmers in Caicó, Rio Grande do Norte, Brazil

Variables	Controls (n = 288) n (%)	Cases (n = 62) n (%)	p-value	Crude OR	95%CI	p-value
Currently employed						
No	80 (27.78)	20 (32.26)	0,479	1.23	0.68-2.23	0.479
Yes	208 (72.22)	42 (67.74)		1.00		
Daily working hours						
< 6	207 (75.82)	39 (70.91)	0.442	1.00	
> 6	66 (24.18)	16 (29.09)		1.28	0.67-2.45	0.443
Employment relationship						
Owner	199 (70.82)	44 (70.97)	0.001	1.00	
Tenant	61 (21.71)	5 (8.06)		0.37	0.14-0.97	0.045
Wage or temporary worker	21 (7.47)	13 (20.97)		2.79	1.30-6.01	0.008
Access to credit						
No	150 (53.96)	24 (40.68)	0,064	0.58	0.33-1.03	0.066
Yes	128 (46.04)	35 (59.32)		1.00		
Indebtedness						
No	158 (56.83)	29 (49.15)	0.281	1.00	
Yes	120 (43.17)	30 (50.85)		1.36	0.77-2.39	0.282
Loss of production						
No	96 (35.04)	25 (43.86)	0.208	1.00	
Yes	178 (64.96)	32 (56.14)		0.69	0.38-1.23	0.210
Exposure to pesticides						
No	177 (65.07)	41 (71.93)	0.320	1.00		
Yes	95 (34.93)	16 (28.07)		0.72	0.38-1.36	0.321
Use of PPE						
No	132 (70.21)	36 (83.72)	0.073	2.18	0.91-5.19	0.078
Yes	56 (29.79)	7 (16.28)		1.00		
Pesticide poisoning						
No	200 (96.15)	39 (84.78)	0.003	1.00		
Yes	8 (3.85)	7 (15.22)		4.48	1.53-13.09	0.006
Stage of farm work						
None	35 (12.82)	10 (17.54)	0.001	1.00		
One or some stages	82 (31.50)	12 (21.05)		0.48	0.19-1.23	0.130
All stages	152 (55.68)	35 (61.40)		0.80	0.36-1.78	0.594

OR = odds ratio; PPE = personal protective equipment.

Additionally, variables with p < 0.20 — access to health services, primary health care (PHC) coverage, use of PPE, and stage of farm work — were included in the multivariable analysis.

In the multivariable model, suicidal behavior showed positive, statistically significant associations with family diagnosis of mental disorder (OR = 2.76), presence of a common mental disorder (OR = 4.25), prior mental health treatment (OR = 2.36), wage or temporary employment (OR = 3.49), and pesticide poisoning (OR = 4.94) (Table 4).

**Table 4:** Multivariable logistic regression of suicidal behavior and health/occupational characteristics among farmers in Caicó, Rio Grande do Norte, Brazil

Variables	Adjusted OR (95%CI)
Health characteristics	
Family diagnosis of mental disorder	
No	Reference
Yes	2.76 (1.16-6.52)
Common mental disorder	
No	Reference
Yes	4.25 (1.63-11.10)
Prior mental health treatment	
No	Reference
Yes	2.36 (1.07-5.19)
Occupational characteristics	
Employment relationship	
Owner	Reference
Tenant	0.16 (0.03-0.77)
Wage or temporary worker	3.49 (1.29-9.37)
Pesticide poisoning	
No	Reference
Yes	4.94 (1.35-18.02)

Pseudo R^2^ = 0.229. OR = odds ratio.

## DISCUSSION

This study identified associations between occupational and health-related factors and suicidal behavior — understood as thoughts of death, suicidal ideation, and suicide attempts — among farmers in the city of Caicó, RN.

With respect to health, particularly mental health, farmers who reported a family history of mental disorder, the presence of a common mental disorder, and prior mental health treatment had higher odds of exhibiting suicidal behavior. These findings are consistent with studies showing an association between suicidal behavior and mental disorders, indicating increased risk among individuals with psychiatric comorbidities.^19^ A global meta-analysis reported an OR of 16.7 for suicide among people with mental disorders.^20^ Another analysis using meta-regression reported ORs ranging from 4 to 8, depending on the psychiatric diagnosis (eg, major depression or dysthymia).^21^

These results underscore the need to reflect on the relationship between prior mental health treatment and suicidal behavior. Farmers who had previously received such treatment were more likely to manifest suicidal behavior. On one hand, this may indicate that treatment identifies individuals with prior diagnoses and specific psychological needs. On the other, it raises questions about the effectiveness of interventions and about the quality, access, and continuity of mental health care available to this population.

Although Brazil’s psychosocial care network — particularly the Psychosocial Care Centers (CAPS, in Portuguese) — has expanded substantially, mental health care in rural areas still faces important limitations. Access remains restricted, and the organization and functioning of the network hinder the effective participation of rural populations in psychosocial care.^22^ Historically, Caicó has undergone significant changes in its mental health landscape and psychiatric reform, beginning with the closure of the psychiatric hospital (Dr. Milton Marinho Psychiatric Hospital) in 2005, which led to the creation of substitute services. The municipality’s mental health network comprises a CAPS III, a CAPS for Alcohol and Drugs, a Specialized Rehabilitation Center III, a clinical center, and a therapeutic residence.^23^

Rural communities have historically faced a lack of resources that would enable them to travel to urban areas, where most health services — especially mental health, social assistance, and education services geared toward the general population — are concentrated.^22^ Moreover, distress in rural areas is tied to low educational attainment, greater dependence on family, and occupational and financial difficulties. The health of rural workers, particularly farmers, is often conditioned by the exploitative nature of agricultural labor and intersects with social, racial, economic, and gender factors. Together, these factors pose substantial challenges for public policies aimed at addressing them.^24^

Another important issue concerns the limited preparedness of health professionals, which persists in most services when identifying and welcoming individuals or family members experiencing psychological distress — especially within PHC. Care is frequently based on a prescriptive complaint-management model, in which health needs are commonly met with referrals to a medical appointment or a specialized level of care.^25^

It is therefore essential to invest in ongoing training for PHC professionals — especially community health agents (CHAs) — so they can adopt less moralistic and more empathetic approaches when interacting with people who have thoughts of death or suicidal behavior. In a study conducted with CHAs in Caicó, training led these professionals to adopt more positive attitudes and to feel better prepared to care for individuals at risk of suicide.^26^

Regarding occupational factors, this study showed that pesticide poisoning nearly quadrupled the odds of suicidal behavior among farmers. Pesticides comprise a set of chemical substances intended to protect crops against pests, diseases, and other harmful organisms. However, improper use, the high toxicity of certain compounds, nonuse of PPE, and weak surveillance and enforcement systems are among the main factors responsible for illnesses and poisonings related to exposure to these substances.^27^ A systematic review reported that pesticide exposure may contribute to the high incidence of depression, anxiety, and suicide among farmers; suicide rates increased in agricultural areas with intensive pesticide consumption, and the included studies showed higher suicide risk among farmers.^4^

Our findings highlight the need to intensify enforcement of pesticide regulations and to strengthen guidance on appropriate use of PPE in agricultural activities, particularly within family farming. In Caicó, agricultural production is largely carried out by smallholders organized as family units. In this context, pesticide use is often embedded in cultural and social practices transmitted across generations, which contributes to neglect of legal norms governing rural labor and worker safety — especially among those in informal employment.^28^ This dynamic is compounded by limited financial resources that often prevent the acquisition and proper use of PPE. As a result, many rural workers use these inputs incorrectly in an effort to increase productivity, even under unsafe conditions.

Accordingly, careful attention should be given to implementing integrated strategies — spanning sectors such as health and education — that ensure access to protective equipment and promote awareness-raising activities about its importance and correct use, thereby helping to mitigate health risks for farmers.

We also observed that farmers with wage or temporary employment contracts were more likely to exhibit suicidal behavior. In a systematic review, Santos et al.^11^ found that aspects of employment relationships — especially seasonal contracts tied to specific periods of the year and sectors — are associated with suicidal behavior in farmers, in contrast to longer-term or fixed wage contracts.

In family farming, seasonal employment relationships are common because climatic variations determine planting and harvesting periods, restricting work to specific times of the year. This seasonality entails precariousness and a lack of financial stability, constituting an important risk factor for mental illness among farmers.

Work-related aspects in agriculture became even more evident during the SARS-CoV-2 (COVID-19) pandemic, when concerns arose about farmers’ financial situation and the potential impact on suicide mortality. Many producers were unable to harvest their crops due to the lack of buyers — either because purchasing was suspended or access to traditional marketplaces was prohibited.^29^ Beyond the economic impacts, social isolation, uncertainty, fear of losing loved ones, and the economic recession heightened people’s vulnerability. This scenario contributed to the onset or worsening of emotional distress and mental health problems, increasing the risk of suicidal behavior.

According to Gunnell et al.,^30^ protective measures can be implemented to reduce psychological distress among agricultural workers. A comprehensive, interdisciplinary response is needed, incorporating selective and universal interventions as prevention strategies. This context also underscores the importance of policies and actions aimed at income generation, employability, and, in particular, support for small businesses, with priority given to family farmers.

Despite the existence of worker-health policies in Brazil, legislation remains limited with respect to mental illness among farmers, and few health actions are directed specifically to this group.

This study has some limitations. There is potential selection bias, as interviews were conducted only with farmers affiliated with STRAAF in the study city. In addition, the data collection instrument was not self-administered due to participants’ difficulty in reading and interpreting the questions, associated with low schooling. Information bias may also have occurred due to recall lapses.

The findings indicate that work-related aspects, pesticide poisoning, and mental health factors are strongly linked to thoughts of death and suicidal behavior among farmers, with associations of considerable magnitude. Accordingly, it is essential to implement public health actions focused on suicide prevention, particularly through individualized care that considers the specificities of family farmers’ health. For these actions to be effective, access to high-quality services must be expanded and improved so that the specific needs of the most vulnerable groups are recognized and addressed, with the goal of reducing suicidal behavior and, consequently, suicide mortality in this population.

The development, strengthening, and implementation of integrated, intersectoral, publicly accountable actions — while not disregarding social responsibility — are crucial for surveillance and suicide prevention among farmers. These strategies should reinforce comprehensive care pathways encompassing health promotion, prevention, treatment, and recovery across all levels of care, ensuring access to the necessary therapeutic modalities.

Additional complementary measures are also important to increase the effectiveness of these actions. These include training and capacity-building for health and education professionals and expanding PHC coverage to enable early recognition of farmers in psychological distress and to facilitate timely reception and referral for treatment. In parallel, actions that promote awareness of correct use of PPE in agricultural work, stronger enforcement, and incentives from federal, state, and municipal authorities also play an important role in reducing suicide in this group.

## CONCLUSIONS

This study found a strong association between occupational factors, pesticide exposure, and mental health conditions and suicidal behavior among farmers in Caicó, RN. Both family history and diagnosis of mental disorders as well as inadequate pesticide use and precarious working conditions — especially seasonal employment — were significant contributors to increased risk of suicidal thoughts, ideation, and attempts in this group.

These results underscore the urgent need for integrated, intersectoral strategies that strengthen comprehensive care for family farmers, prioritizing promotion, prevention, treatment, and rehabilitation across all levels of care. It is also essential to expand access to appropriate mental health services; train and upskill professionals — particularly those in PHC and CHAs; and implement educational and enforcement actions regarding correct use of PPE.

Finally, public policies that support income generation, job stability, and small rural enterprises are fundamental to mitigating vulnerabilities linked to psychological distress and suicidal behavior in this population. Such measures are expected to help reduce suicide among farmers and promote health and quality of life in rural settings.
